# High-Precision Automated Soybean Phenotypic Feature Extraction Based on Deep Learning and Computer Vision

**DOI:** 10.3390/plants13182613

**Published:** 2024-09-19

**Authors:** Qi-Yuan Zhang, Ke-Jun Fan, Zhixi Tian, Kai Guo, Wen-Hao Su

**Affiliations:** 1 College of Engineering, China Agricultural University, Beijing 100083, China; 2 Yazhouwan National Laboratory, Sanya 572000, China; 3 Institute of Environment and Ecology, Shandong Normal University, No. 88, Wenhuadong Road, Lixia District, Jinan 250014, China

**Keywords:** phenotype acquisition, soybean phenotypes, instance segmentation, smart agriculture

## Abstract

The automated collection of plant phenotypic information has become a trend in breeding and smart agriculture. Four YOLOv8-based models were used to segment mature soybean plants placed in a simple background in a laboratory environment, identify pods, distinguish the number of soybeans in each pod, and obtain soybean phenotypes. The YOLOv8-Repvit model yielded the most optimal recognition results, with an $R^{2}$ coefficient value of 0.96 for both pods and beans, and the RMSE values were 2.89 and 6.90, respectively. Moreover, a novel algorithm was devised to efficiently differentiate between the main stem and branches of soybean plants, called the midpoint coordinate algorithm (MCA). This was accomplished by linking the white pixels representing the stems in each column of the binary image to draw curves that represent the plant structure. The proposed method reduces computational time and spatial complexity in comparison to the A* algorithm, thereby providing an efficient and accurate approach for measuring the phenotypic characteristics of soybean plants. This research lays a technical foundation for obtaining the phenotypic data of densely overlapped and partitioned mature soybean plants under field conditions at harvest.

## 1. Introduction

Soybeans [*Glycine max* (L.) Merr.] are cultivated worldwide due to their high protein and oil content, which are beneficial for human and animal consumption [1,2]. Furthermore, the seed of soybeans is high in protein and oil content, resulting in uses as human food, animal feed, and industrial products [3]. In recent years, there have been notable improvements in soybean yields, largely due to the dedicated efforts of soybean breeders [4]. However, there is still a need for the development and breeding of new soybean varieties that exhibit high yields, high quality, and multiple resistances [5]. The plant breeding process requires the phenotyping of a vast array of populations [6]. Improving soybean yield and quality is the focus of breeding efforts [7]. However, due to the complexity of soybean plants, research on extracting phenotype parameters and analyzing growth conditions based on plant image models is still limited. The most commonly used method is still traditional manual measurement, but this method is time-consuming and laborious [8]. An automated recording of phenotypic traits allows for the rapid and inexpensive collection of genetic diversity data, thereby increasing the efficiency of selection for these traits in breeding programs. Therefore, it is critical to have reliable and accurate phenotypic estimates of soybean at maturity. This is necessary for predicting yield and losses in soybean, as well as for assessing the merit of crop germplasm.

Computer vision has been used extensively in agriculture, particularly in crop phenotyping for forecasting and yield prediction [9,10,11,12,13]. For instance, Weyler, et al. [14] proposed a vision-based method to segment individual crop leaves and associate each leaf with its corresponding crop plant in a real field. This is a novel approach for computing clustered regions based on network predictions, which achieves high accuracy. As deep learning evolves, an artificial intelligence and multispectral image processing system using convolutional neural network (CNN) algorithms for leaf segmentation has been developed [15]. The solution calculates vegetation indices (VI) with high accuracy and spatial resolution, enabling the remote assessment of plant health and the identification of stressed plants. Later, the instance segmentation model and the RGB video captured by the UAV were used to measure the phenotypic characteristics of the detected bunches and berries at an early stage [16]. A comparison of two instance segmentation algorithms, YOLACT and Spatial Embeddings, with respect to the number of berries detected per bunch showed that Spatial Embeddings evaluated the number of berries more accurately (79.5%) than YOLACT (44.6%). Nevertheless, there is a paucity of research on instance segmentation of soybean plants using CNNs, and there is a notable dearth of advanced algorithms for efficiently separating the main stems and branches of soybean plants.

In recent years, the combination of deep learning-based image segmentation algorithms with agriculture has facilitated the development of smart agriculture [17,18], using Mask R-CNN and transfer learning to phenotype high-throughput soybean seeds for soybean phenotyping. They achieved effective segmentation of individual soybean seeds and efficient computation of morphological parameters for each seed. This approach is practical for high-throughput object instance segmentation and high-throughput seed phenotyping. However, the computational cost of this study is high [19]. Yu et al. [20] proposed a high-throughput phenotyping method based on unmanned aerial vehicle (UAV) systems. This method can be used to monitor and quantitatively characterize soybean canopy development in different genotypes. They employed an infrared feature fusion segmentation network (RIFSeg-Net), which demonstrated superior performance compared to a conventional deep learning image segmentation network (precision = 0.94, recall = 0.93, and F1 score = 0.93). The SPP-extractor (soybean plant phenotype extractor) algorithm was proposed for soybean phenotypic traits [7]. An attention mechanism for target detection has been added to the standard YOLOv5s model. A branch recognition and measurement module were designed, combining image processing, target detection, semantic segmentation, and heuristic search. The final experimental results on real plants showed that the $R^{2}$ scores of the SPP extractor on the four plants were between 0.93 and 0.99. While the research yielded superior outcomes, the employed models were outdated, and the separation algorithms exhibited considerable scope for enhancement.

This study presents a novel methodology that integrates CNNs with algorithms for the separation of the main stem and meristem of soybean plants. This approach enables the prediction of the phenotype of mature soybean plants in a straightforward manner within a laboratory setting. It establishes the technical basis for the subsequent phase of predicting soybean phenotypes in complex field settings. The primary objectives are outlined below: (1) propose an enhanced YOLOv8 framework for instance segmentation of soybean plants; (2) introduce various improvements to the performance of the YOLOv8 model for identifying pods; (3) conduct further image processing on segmented pods and extracted soybean plants to obtain images containing only the main stem and branches; (4) present an effective algorithm called the midpoint coordinate algorithm (MCA) for the separation of the main stem and branches of soybean plants and the independent calculation of their lengths; and (5) introduce an innovative processing technique involving the drawing of curves representing the plant structure by connecting white pixels representing the stem in each column of the binary image.

## 2. Results

### 2.1. Model Training

**Figure 1:**
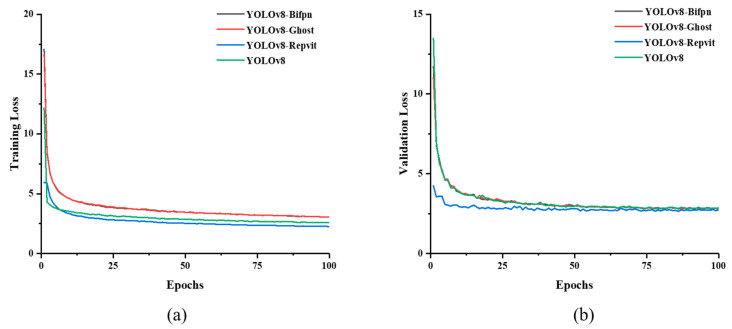
Recognizing training loss curves (**a**) and validation loss curves (**b**) for pods and beans using 4 proposed YOLOv8-based models.

Four different CNN-based models were developed for pod and bean labeling in images. Figure 1a shows the training loss trends for pod and bean grain recognition. The results indicate that as the number of epochs increases, all models display a gradual reduction in the loss curve. Among them, YOLOv8-Repvit achieves the lowest loss value and stabilizes after 50 epochs. While YOLOv8 shows the fastest rate of convergence, its associated loss value is the second lowest, stabilizing after 60 epochs. The loss trends for YOLOv8-Bifpn and YOLOv8-Ghost are similar, demonstrating comparable performance but also the highest loss values. Taken together, YOLOv8-Repvit has the most superior performance.

Figure 1b presents the validation loss trends for pod and bean identification, which appear similar to Figure 1a. YOLOv8-Repvit consistently shows the lowest loss values and the fastest convergence. However, the other three models show a generally consistent trend, all gradually stabilizing after 50 epochs. These results demonstrate that the three proposed enhancements to YOLOv8 improve the model’s accuracy, reduce complexity, and achieve optimal convergence speeds, with YOLOv8-Repvit showing the most favorable performance.

### 2.2. Overall Performance of Modified YOLOv8

**Figure 2:**
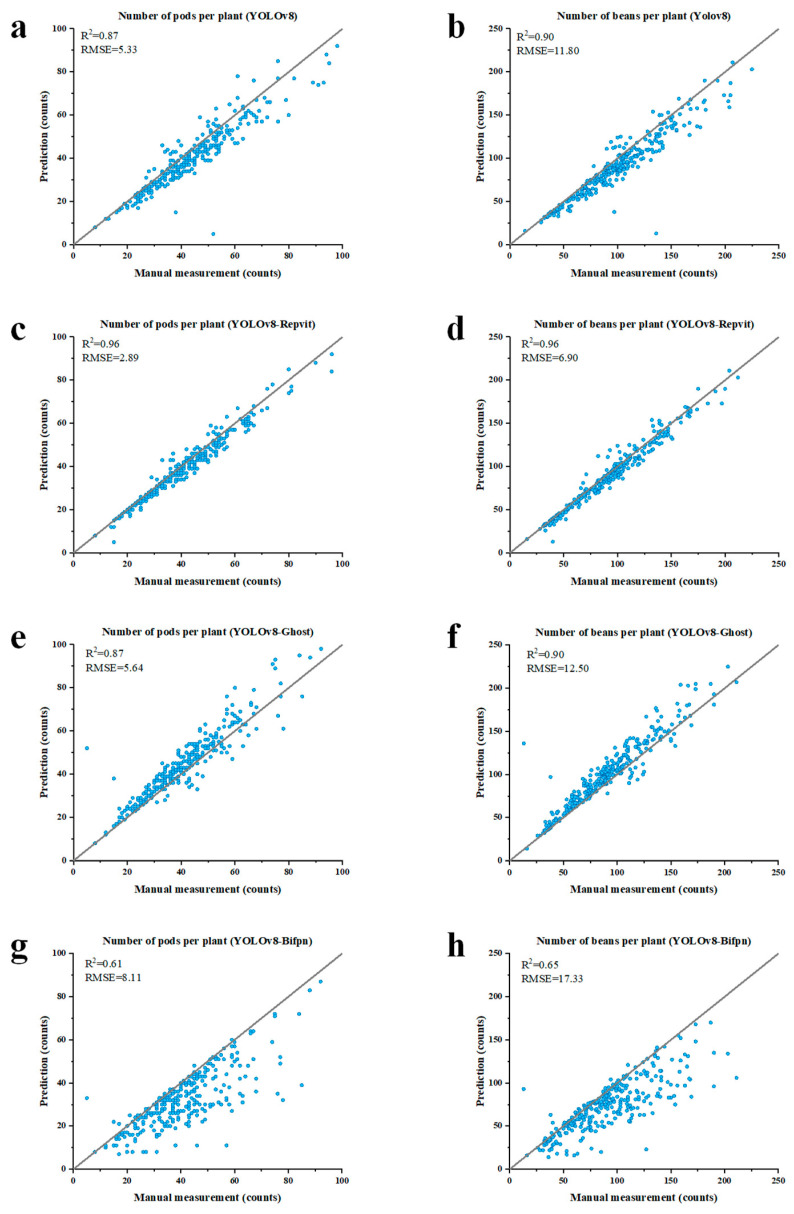
Correlation analysis of four proposed YOLOv8-based models against different soybean phenotypes predicted and counted: (**a**,**b**) Correlation analysis between YOLOv8 and manual counting of pod and bean numbers. (**c**,**d**) Correlation analysis between YOLOv8-Repvit and manual counting of pod and bean numbers. (**e**,**f**) Correlation analysis between YOLOv8-Ghost and manual counting of pod and bean numbers. (**g**,**h**) Correlation analysis between YOLOv8-Bifpn and manual counting of pod and bean numbers.

The number of pods per plant was determined using four models based on YOLOv8. A total of 545 soybean strains were used, with thousands of images captured in a laboratory setting to evaluate the efficacy of the proposed models. As shown in Figure 2, this is comparable to human performance. The YOLOv8 model achieved an $R^{2}$ coefficient of 0.87 and a root mean square error (RMSE) value of 5.33. The $R^{2}$ coefficient was further improved when the same extraction was performed using the enhanced YOLOv8 model. As shown in Figure 2c, the YOLOv8-Repvit model improved the $R^{2}$ coefficient up to 0.96. Meanwhile, the RMSE value was reduced to 2.89. In plants with numerous pods, the pods tend to be densely packed, with some pods obscured by other pods and stems. This poses a challenge to the modeling effect, as evidenced by the point deviating from the model line in the lower left corner of Figure 2a. The improved model, however, significantly mitigates this issue, as shown in Figure 2c, where all points are more centered near the 1:1 line. Conversely, the YOLOv8-Bifpn model exacerbates the issue, with a substantial increase in points below the 1:1 line, as illustrated in Figure 2g.

**Figure 3:**
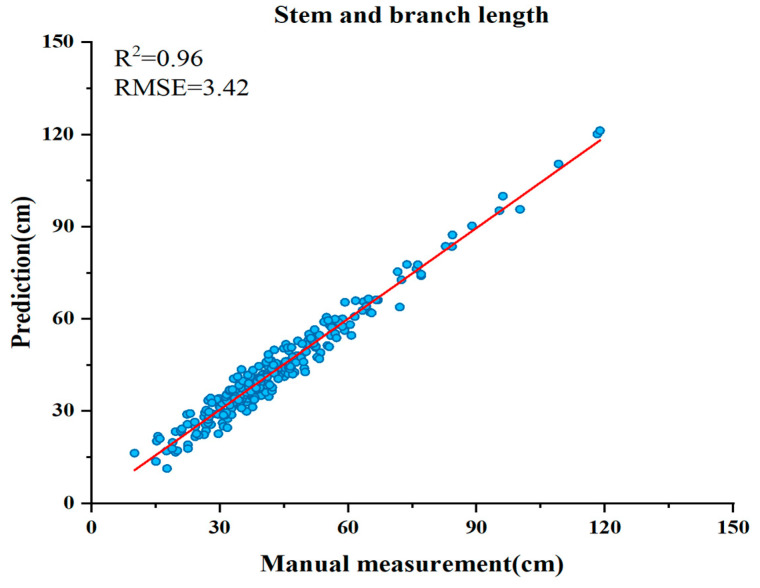
Correlation analysis of midpoint coordinate algorithm (MCA) against stem and branch length predicted and measured.

A similar outcome was observed when the number of beans per plant was extracted. Figure 2b shows that the YOLOv8 $R^{2}$ coefficient is 0.90 and the root mean square error value is 11.80. Furthermore, Figure 2f illustrates that the deployment of YOLOv8-Ghost yields outcomes that are analogous to those of the original model with an $R^{2}$ coefficient of 0.90 and an RMSE value of 12.50. Figure 2d shows that extraction with YOLOv8-Repvit significantly reduces the deviation from the model line, achieving an $R^{2}$ coefficient value of 0.96 and an RMSE value of 6.90. Conversely, Figure 2h shows that YOLOv8-Bifpn exacerbates the same situation. Figure 3 shows that the proposed method for stem and branch length extraction achieved an $R^{2}$ coefficient of 0.96 and an RMSE value of 3.42. The results demonstrate that both YOLOv8-Repvit and the MCA proposed in this study exhibit superior performance.

The results of the correlation analysis are summarized in Table 1. Among the four YOLOv8-based models, YOLOv8-Repvit exhibited the highest $R^{2}$ of 0.96 and the smallest RMSE compared to the other three models, irrespective of whether the predictor was number of pods per plant or number of beans per plant. As illustrated in Table 2, the mean prediction time for all models is approximately 7.8 ms, with minimal discrepancies between models. The YOLOv8-Repvit model exhibits the highest box (mAP50) and mask (mAP50) values among the YOLOv8 series, at 0.857 and 0.856, respectively. These values represent an improvement of 0.009 and more over the original YOLOv8 model. The results demonstrate that this model markedly enhances accuracy without a substantial increase in processing time (only 0.1 ms). The box (mAP50) and mask (mAP50) values of YOLOv8-Ghost and YOLOv8-Bifpn are also improved, but to a lesser extent than YOLOv8-Repvit, and the processing time is longer than that of YOLOv8. Consequently, YOLOv8-Repvit exhibits the most optimal overall performance.

### 2.3. Comparison Results of Target Detection Models

**Figure 4:**
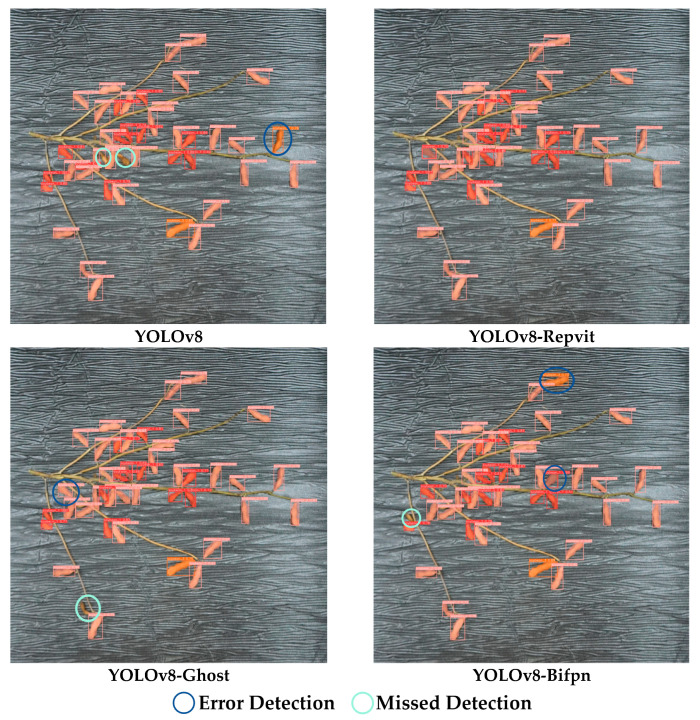
Comparison of pod and bean recognition results for four YOLOv8-based models: red, pink, and orange boxes represent the identification of pods with one, two, and three beans, respectively; blue circles indicate misidentifications, while green circles denote missed identifications.

Figure 4 illustrates the results of four YOLOv8-based models used to identify soybean phenotypes. The red, pink, and orange boxes indicate that the models identified pods containing one, two, and three beans, respectively. The blue circles indicate recognition errors, and the green circles indicate that the target was not recognized.

In the case of the same soybean sample image, the YOLOv8 model exhibited the poorest recognition performance, with two instances of missed detection and one instance of erroneous detection. YOLOv8-Bifpn exhibited three errors: one missed detection and two erroneous detections. YOLOv8-Ghost had one missed detection and one erroneous detection. In contrast, YOLOv8-Repvit demonstrated the highest accuracy, with no errors in recognition. Therefore, YOLOv8-Repvit’s recognition performance is also the most superior in situations where samples are actually recognized.

## 3. Discussion

This study utilized a modified YOLOv8 model to segment soybean plants, identify pods, and count the number of soybeans within each pod. Following this, additional image processing was performed on the segmented pods and extracted soybean plants, producing images that depicted only the main stems and branches. This facilitated the precise measurement of the lengths of the main stems and branches. The YOLOv8-Repvit model exhibited superior performance in segmentation and recognition. Its high versatility enables multiple tasks to be completed in a single run, thereby reducing computation time and enhancing overall efficiency. Additionally, an algorithm named MCA was proposed to effectively separate the soybean main stems and branches, allowing their lengths to be calculated independently. The plant’s structure is represented by a series of curves, each of which connects the white pixels that signify the stems in each column of the binary image. This technique enables the calculation of the lengths of the main stem and branches. In comparison to the conventional A* algorithm, MCA presents a notable improvement in terms of reducing computational time and spatial complexity, thereby offering an efficient approach to accurately measure the phenotypic characteristics of soybean plants. Although the model demonstrated efficacy in identifying soybean phenotypic data, it exhibited some limitations in accurately quantifying the number of bean grains. Further research should concentrate on developing the algorithm’s capacity to differentiate between finer details and fuzzy areas [21].

As shown in Table 3, in studies that did not use YOLOv8-based crop phenotyping acquisition, a novel automated corn tassel phenotyping system called corn tassel segmentation (CTS) was developed for identifying the length of crop branches [22]. The CTS employs a PointNet++ network to train and learn the tip features of the point cloud, enabling the automatic segmentation of the tassel branch tip point cloud. The complete branch segmentation is achieved using a shortest path algorithm. The squared $R^{2}$ of branch length is 0.99, indicating a high degree of accuracy. However, this study only experimented with spikes that could be quickly labeled manually and could not handle phenotypes with complex edges. For potatoes, Songtao et al. [23] used OCRNet to semantically segment the top view RGB image of potatoes. The accuracy of three representative and easy-to-measure phenotypic parameters, leaf number, plant height, and maximum width was assessed, and the coefficient of $R^{2}$ was 0.93, 0.95, and 0.91, respectively, which was lower than the $R^{2}$-value in the proposed method. For the soybean, Zhou et al. [7] proposed the SPP extractor. This unified algorithm is designed to automatically extract four phenotypic characteristics of soybean plants at harvest time. The SPP extractor can accommodate extensive overlap between pods and stems, eliminating the need for prior removal of pods from the plant. This augmentation of the YOLOv5s model with the SE attention mechanism enhances its performance. The $R^{2}$ scores of the SPP extractor on pod number per plant, plant height, effective branch number, and branch length were 0.93–0.99, respectively. In addition, the YOLOv5 series of models is now somewhat outdated. Moreover, the A* algorithm used takes a long time to compute.

Part of the research on crop phenotype acquisition using YOLOv8-based models is also shown in Table 3. Sapkota et al. [24] combined the YOLOv8 object detection algorithm and geometric fitting techniques to 3D point cloud data to successfully achieve accurate size measurements of immature green apples. However, the $R^{2}$ value of Realsense D435i was low at 0.77. When Guan et al. [25] used the improved YOLOv8 model, DBi-YOLOv8, to detect various phenotypes of maize, with an $R^{2}$ of 0.93 for leaves and 0.92 for tassels in the canopy, the canopy was 0.92. The application of the YOLO series of models to soybean phenotypes, particularly pod and stalk length, has been relatively understudied. He et al. [26] employed the enhanced YOLOv5 model to predict the single pod weight of soybean and achieved an R-value of 0.95. While the experimental results are promising, they do not provide sufficient data for the research of soybean breeding and yield prediction. Subsequently, Lu et al. [27] employed an enhanced YOLOv3 model to accurately identify soybean pods, achieving 90.3% accuracy. The studies on soybean yielded a single phenotype. It is not feasible to obtain and study comprehensive information regarding soybean phenotypes, and the identification results are exceedingly limited and not comprehensive. Moreover, no study has yet employed the YOLO series of models for the acquisition of soybean stem phenotypes.

In contrast, the latest YOLOv8-Repvit model utilized in this paper outperformed compared to the above studies. Not only can it accurately segment the edges of pods and backgrounds, but it can also obtain more complete soybean phenotypic information. YOLOv8-Repvit was used to segment densely overlapping mature soybean plants at harvest. This was conducted to identify pods, distinguish the number of soybeans in each pod, and obtain soybean phenotypes. The $R^{2}$ coefficients reached 0.96. Additionally, an algorithm was proposed to efficiently separate main stems from branches of soybean plants. This algorithm also reached 0.96 in the $R^{2}$ coefficient, indicating that the method can extract soybean phenotypes with greater accuracy and efficiency. Compared with the A* algorithm, the proposed method reduces the computation time and space complexity. Overall, compared with the study based on YOLOv8, the experimental results of this paper all have $R^{2}$ coefficient values of 0.96, which are greater than those of the studies. Compared with other models, the $R^{2}$ coefficient of the proposed method is higher than most of the studies in Table 3. This indicates that the YOLOv8-based improvement in this study possesses superior performance.

**Table 3:** Comparison of studies on crop phenotype acquisition based on different deep learning methods.

References	Crop	Model	Extracted Phenotypes	Evaluation Parameters	Value
Zermas et al. [28]	Corn	A 3D model and point cloud processing algorithms	The individual plant segmentation and counting	Accuracy	88.1%
			Leaf area index (LAI)		92.5%
			The individual plant height computation		89.2%
			The leaf length extraction		74.8%
Zhang et al. [22]	Corn	PointNet ++ [29]	Branch length	*R* ^2^	0.99
			Branch angle		0.94
			Branch count		0.96
Zhu et al. [30]	Tomato	Mask R-CNN	Fruit color, horizontal and vertical diameters, top and navel angles, locule number, and pericarp thickness	Accuracy	95.0%
Songtao et al. [23]	Potato	OCRNet [11]	Leaf number	*R* ^2^	0.93
			Plant height		0.95
			Maximum width		0.91
Zhou et al. [7]	Soybean	The SPP extractor (soybean plant phenotype extractor)	Pod number per plant, plant height, effective branch number, branch length	*R* ^2^	0.93–0.99
He et al. [26]	Soybean	Improved YOLOv5	Single pod weight	*R* ^2^	0.95
Lu et al. [27]	Soybean	Improved YOLOv3	Pods	Precision	90.3%
Xiong et al. [31]	Rice	Panicle-SEG-CNN	Rice panicle	Precision	82.1%
Teramoto and Uga [32]	Rice	U-Net [33]	The vertical centroid of root distribution	*R* ^2^	0.99
			The horizontal centroid of root distribution		0.96
Yu et al. [34]	Lettuce	2DCNN, FCNN, Deep2D and DeepFC	The soluble solid content (SSC)	*R* ^2^	0.90
			pH		0.85
Zhang et al. [35]	Strawberry	HR-YOLOv8	Strawberry ripeness	Precision	92.3%
Yu et al. [20]	Strawberry	CES-YOLOv8	Strawberry ripeness	Precision	88.2%
Orchi et al. [36]	13 different plant species	YOLOv8	Crop leaf disease	Precision	63.3%
Sapkota et al. [24]	Apple	YOLOv8	Azure Kinect	*R* ^2^	0.90
			RealSense D435i		0.77
Guan et al. [25]	Corn	DBi-YOLOv8	Leaves	*R* ^2^	0.93
			Tassels in the canopy		0.92
Proposed method	Soybean	YOLOv8-Repvit	Pods	*R* ^2^	0.96
			Beans		0.96
		MCA	Stem and branch length		0.96

The findings of this study illustrate the potential of the proposed methodology for the acquisition of soybean phenotypes. The methodology enables the identification of individual pods and the enumeration of the number of soybeans within each pod. Furthermore, the method can ascertain the length of the main stem and branches. It should be noted, however, that this study represents a preliminary investigation, and the images that were processed were of soybeans placed in a simple background in a laboratory environment. Further experimentation is required to assess the efficacy of the method in a field environment. The accuracy of recognizing pods and obtaining the length of stems is contingent upon the accuracy of the proposed model, as well as the clarity of the image [37,38]. Furthermore, pod shading affects the accuracy of soybean population prediction [39], suggesting that accurate pod counting is challenging [9,40]. The subsequent phase of this research will address the handling of occlusion situations and the processing of images in complex backgrounds. Furthermore, portable devices were developed. The images captured by the device’s RGB camera will be wirelessly transmitted to a microcomputer for subsequent analysis. After the modeling phase, the real-time acquisition of soybean phenotypic characteristics will facilitate the estimation of soybean growth and prediction of yield. A soybean phenotype remote detection and advisory system was planned with the objective of employing advanced deep learning and image segmentation techniques to facilitate broader applications in the field of agriculture. Moreover, the methodology for differentiating between main stems and branches can be further refined to improve efficiency and accuracy. The implementation of related research will significantly enhance the efficiency and accuracy of soybean phenotype acquisition, thereby facilitating the rapid and cost-effective collection of genetic diversity data [41]. This will, in turn, enhance the efficiency of breeding programs for the selection of soybean traits [42].

## 4. Materials and Methods

### 4.1. Sample Preparation

The soybean varieties used in this study were sourced from Dongying City, Shandong Province, with cultivation beginning in June and concluding with harvest in September. To achieve the objectives of the breeding study, a comprehensive phenotypic analysis was conducted on various soybean varieties. The imaging platform employed a Sony α6400 camera (Sony Corporation, Tokyo, Japan), mounted on a central stand, with a resolution of 6000 × 4000 pixels. Images were captured in JPG format. The camera was connected to a computer via a USB cable, allowing for control over shooting parameters, real-time preview, and image capture. This setup ensured an automated and efficient photographic process throughout this study.

To optimize the imaging of soybeans, a specialized photography platform was developed, consisting of a robust metal frame and a black base plate centrally positioned. The base plate, coated with adhesive, stabilized the soybean stalks and provided contrast against the background. The frame was enclosed with a green shade cloth, and a top-mounted LED light tube was used to eliminate external light interference, ensuring consistent and uniform illumination. Before imaging, meticulous preparation of the soybean samples was conducted to maximize image clarity and accuracy. Leaves and soil were carefully removed from the stalks and pods to isolate the key structural components of the plant. The stalks and pods were then evenly arranged on the adhesive-coated base plate to minimize overlap between plant parts, ensuring that each section of the plant could be distinctly captured. This arrangement not only improved the visibility of the pods and stems but also facilitated more accurate segmentation during image analysis.

To ensure precise measurements of plant characteristics, a ruler was attached to the bottom edge of the camera platform. This ruler served as a reference scale in every image, allowing for the conversion of pixel dimensions into actual physical measurements. By incorporating this reference, the imaging system enabled accurate extraction of plant height, pod size, and other key phenotypic traits. This carefully designed setup ensured reliable data acquisition and contributed to refining methods for the analysis of soybean phenotypic characteristics.

### 4.2. Data Acquisition

**Figure 5:**
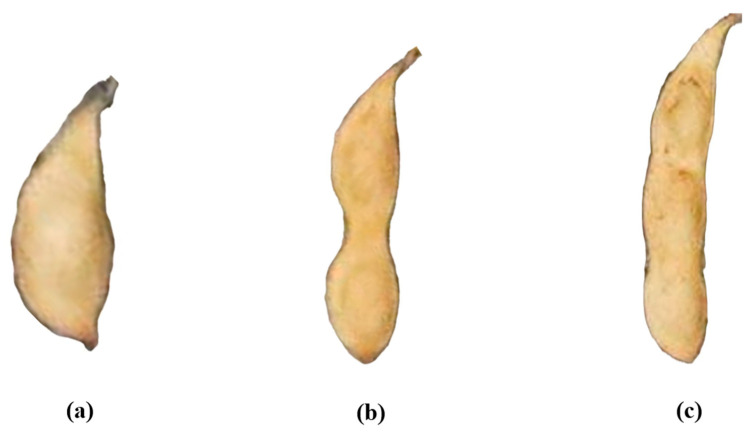
Images of pods containing different numbers of beans: (**a**) pod with one bean; (**b**) pod with two beans; and (**c**) pod with three beans.

**Figure 6:**
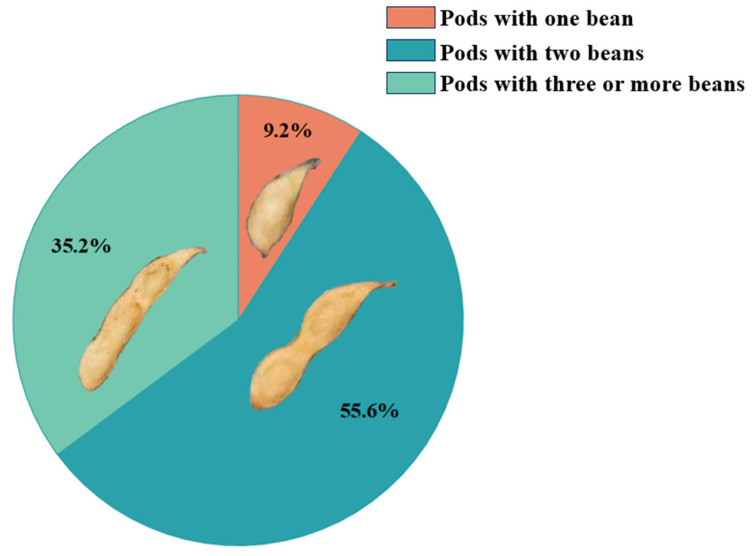
Distribution of the number of different pod forms is shown: red partitions indicate pods containing one bean, blue partitions indicate pods containing two beans, and green partitions indicate pods containing three or more beans.

For this study, the soybean pods were categorized based on the number of beans they contained: one, two, or three. Pods containing four or more beans were extremely rare, constituting less than 0.5% of the total sample. To avoid negatively impacting the training performance due to data imbalance, these pods were grouped under the “three-bean” category. Figure 5 visually presents the different pod morphologies used in this study. The total number of labeled pods was 12,110. After data augmentation, this number increased to 60,550 pods. The total number of labeled plants was 442 for training and validation of the four YOLOv8-based models. The dataset in this study is sufficient in both sample size and diversity to meet the requirements of the soybean pod recognition task. Figure 6 illustrates the distribution of the number of different pod morphologies, with red partitions indicating pods containing one bean, blue partitions indicating pods containing two beans, and green partitions indicating pods containing three or more beans. Most of the pods (55.6%) were 2-seeded, followed by 3-seeded and 1-seeded pods.

Images of all the desired strains were taken in the field, totaling several thousand. For one of the strains, multiple soybean plants were photographed. Ultimately, we selected a few hundred photos for this study. Among these, 442 images were used specifically for training and validating the YOLOv8 model.

To improve the generalization capability of the YOLOv8-based models, various data augmentation techniques were applied to the dataset. These techniques included adjustments to brightness, horizontal flipping, noise addition, and image translation, all of which helped diversify the training data and mitigate overfitting. The augmentation process expanded the total dataset to 2210 images, enhancing the variability and robustness of the training samples. From this augmented dataset, 1545 images (70%) were allocated for training the model, while 665 images (30%) were reserved for validation. This balanced split ensured a reliable evaluation of the model’s performance while preventing any potential biases in the training process.

Additionally, 200 images of soybean plants were captured in the laboratory to evaluate four different models based on YOLOv8, including methods for extracting stem and meristem lengths. Table 4 provides an overview of the dataset used in the experiment, detailing the different stages and volumes of data used for model development and testing.

The formula for calculating the total number of beans is shown below:

(1) $$N_{total}=N_{1}\times 1+N_{2}\times 2+N_{3}\times 3,$$

where $N_{total}$ is the total number of soybean seeds, $N_{1}$ is the total number of pods containing one seed each, $N_{2}$ is the total number of pods containing two seeds each, and $N_{3}$ is the total number of pods containing three seeds each.

### 4.3. Main Methods

**Figure 7:**
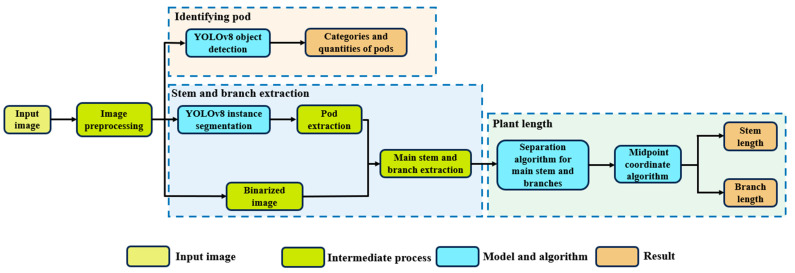
The research flowchart of this study: three modules consisting of pod region identification, stem and branch extraction, and branch length calculation.

The objective of this study was to collect data on the phenotypic characteristics of soybean plants. The focus was on the statistical analysis of the number of soybean pods and seeds, as well as the measurement of their stem length and branch length. To achieve this goal, a series of workflows was constructed as shown in Figure 7. The first step in the process involves the preliminary processing of input images to ensure that they are suitable for analysis. Once this step is completed, the YOLOv8 algorithm is employed to detect soybean pods, followed by the classification and enumeration of the identified objects. Pod extraction is carried out using YOLOv8 instance segmentation, and the resulting binary images are used for the subsequent extraction of the main stem and branches. The length of the plant is determined by analyzing the main stem and branches using a separation algorithm, and specific values are calculated using the centroid method. This approach facilitates the accurate acquisition of phenotypic data on the length of soybean stems and branches.

**Figure 8:**
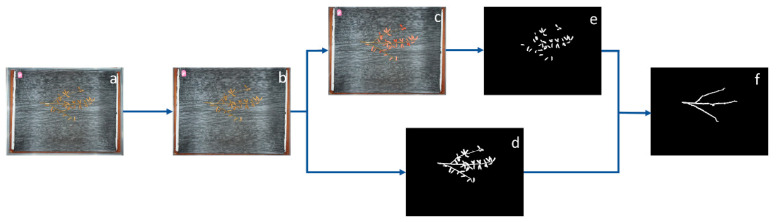
The process of specific phenotype collection: original image (**a**), image after reduction in distortion (**b**), image after instance segmentation (**c**), image after binarization of pods and stems (**d**), image after binarization of pods (**e**), and image after stem extraction (**f**).

The phenotypic data acquisition process is illustrated in Figure 8, detailing each step from initial image capture to final feature extraction. The first step is image correction, which addresses irregular external boundaries and non-standard shapes caused by the camera’s shooting angle and lens distortion. Figure 8a represents the original image as captured, while Figure 8b shows the corrected image. The geometrical distortions, primarily due to perspective and lens effects, are rectified to ensure that the measurements correspond to actual plant dimensions. This correction step is critical in maintaining the accuracy of phenotypic data. Additionally, during this phase, only the area of the base plate—representing the relevant camera shot—is retained for analysis, ensuring that only the useful portions of the image are considered. Once the images are corrected, the next stage focuses on pod instance segmentation and binarization, as shown in Figure 8b–d. Instance segmentation is applied to separate the pods from the rest of the image, allowing for their clear identification and classification. Concurrently, the process of binarization is performed on both the pods and stems, converting the segmented images into black-and-white representations that simplify further analysis. Figure 8c depicts the results of instance segmentation, while Figure 8d shows the binarized image of both pods and stems. To further refine the analysis, the pods are isolated from the rest of the plant by performing additional binarization, as illustrated in Figure 8e. This step is crucial in ensuring that the subsequent measurements of the main stem and branches are accurate and not influenced by pod regions. The resulting images provide clear distinctions between different plant structures, facilitating more precise extraction of the relevant features. Following the binarization of the pods and stems, the final step in the workflow involves extracting the soybean stalks and calculating the lengths of the main stem and branches. This process is depicted in Figure 8d–f. A separation algorithm is employed to distinguish the main stem from the branching structures, after which the lengths of both are measured. The MCA is used to calculate the specific lengths of the stems and branches, providing quantitative data on the structural features of each soybean plant. This approach ensures that all phenotypic measurements are highly accurate and consistent across the dataset.

#### 4.3.1. Pod Identification and Counting

The detection of soybean pods is achieved through the application of target detection and instance segmentation algorithms. To improve accuracy, soybean pods are first separated to reduce overlap during the imaging process. Concurrently, instance segmentation of soybeans is conducted to achieve more accurate recognition of soybean plants.

**Figure 9:**
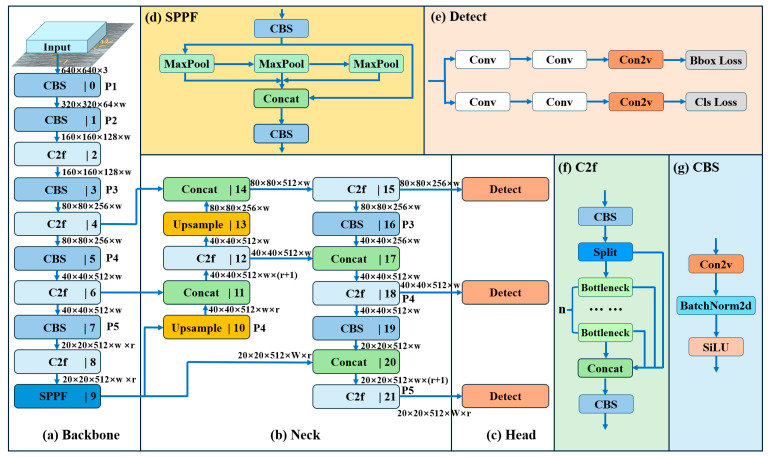
YOLOv8 network structure diagram: Backbone (**a**), Neck (**b**), Head (**c**), SPPF (spatial pyramid pooling—fast) (**d**), Detect (**e**), C2f (**f**), and CBS (**g**).

YOLOv8 is a widely recognized neural network for instance segmentation tasks, based on the development of the YOLO (you only look once) model [43]. In comparison to its predecessors, such as YOLOv5 and YOLOv7 [44], YOLOv8 offers significant improvements in detection accuracy and speed, mainly due to the new backbone network, anchorless detection header, and innovative loss function. Figure 9 provides a description of the network structure of YOLOv8. Its backbone network combines CSP and ELAN concepts with lightweight C2f modules to enhance gradient flow [45]. YOLOv8 also introduces varifocal loss and focal loss to optimize the model performance and uses the SPPF module in the final stage to maintain the accuracy of multi-scale detection. These improvements enable YOLOv8 to excel in object detection tasks in computer vision [23,46].

To improve the phenotyping of soybean plants, the following modifications have been made based on YOLOv8: Repvit [47], Ghost [48], BIFPN [49].

Transformer-based models, such as SwinTransformer [50], EfficientViT [51], and RepVit, have shown good performance in complex scenes and small object detection [52]. The RepViT model, introduced in 2023, marks a departure from conventional lightweight CNNs by embracing the MetaFormer block structure [53], predominantly featured in lightweight Vision Transformers (ViTs). Consequently, RepViT achieves notable enhancements in performance and efficiency, surpassing existing benchmarks set by the leading lightweight ViTs and CNNs in the field.

**Figure 10:**
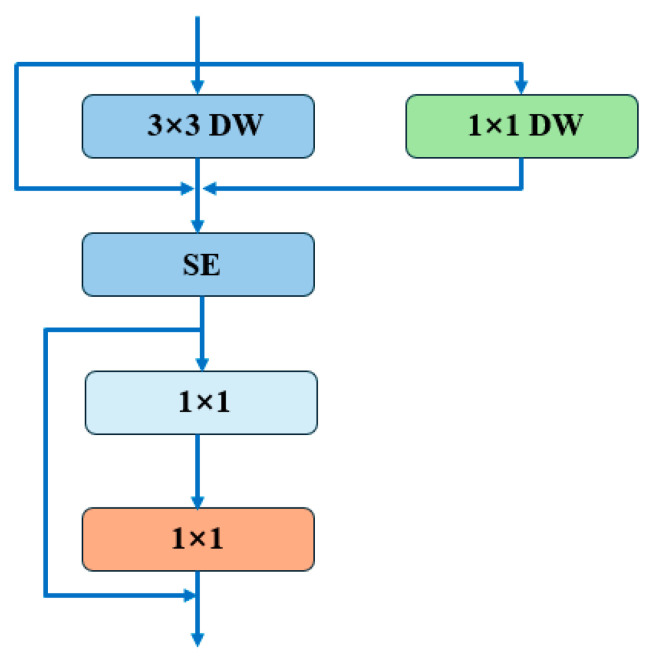
RepViT architecture.

The RepViT architecture employs structural re-parameterization to unify distinct token and channel mixers within its block design, effectively decreasing latency while either preserving or enhancing performance metrics. On a finer scale, the selection of kernel dimensions and the strategic positioning of Squeeze-and-Excitation (SE) layers [54] are meticulously calibrated to optimize the trade-off between computational efficiency and model effectiveness. During inference, this design consolidates the multi-branch topology into a singular, deep convolutional layer, thereby mitigating the computational overhead typically incurred by network de-connectivity. The structure of the module is shown in Figure 10 [47].

Ghost convolution represents a sophisticated technique in the domain of deep neural networks (DNNs), offering enhanced efficiency in feature extraction through the partitioning of the conventional convolutional layer into two discrete phases. In the initial phase, a set of streamlined feature maps is generated through the application of conventional convolution. In the second phase, the feature maps are expanded through a series of simple linear operations, resulting in what are termed ghost feature maps. The Ghost module replicates feature information at an equivalent level with a markedly reduced number of parameters, thereby effectively capturing the similarities and critical redundancies among the network’s feature layers. This approach markedly diminishes the consumption of computational resources while ensuring the effective extraction of information and the maintenance of the model’s overall performance.

**Figure 11:**
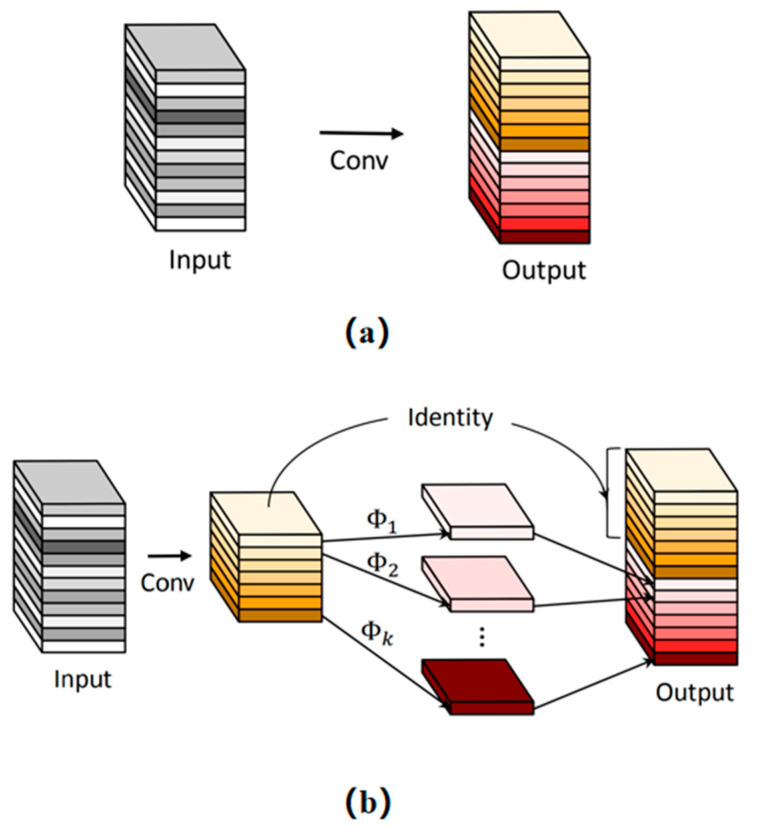
The structure of Ghost: (**a**) The convolutional layer and (**b**) The Ghost module [48].

As illustrated in Figure 11, the process begins with the generation of a subset of original feature layers through standard convolution operations. Subsequently, each channel of these original feature layers is subjected to depth wise convolution (DW) processing to generate the ghost feature layers. Finally, by concatenating the original feature layers with the ghost feature layers through a Concat operation, a complete output feature layer is formed, embodying an elegant balance between accuracy and efficiency in model design [48].

**Figure 12:**
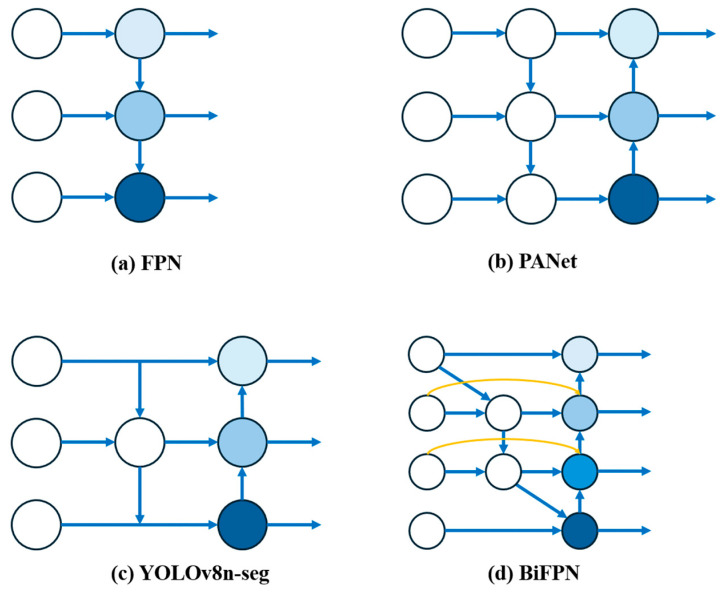
Four different neck network architectures: FPN (**a**), PANet (**b**), YOLOv8n-seg (**c**), and BiFPN (**d**), as the color of the blue nodes deepens, the semantic information gradually increases.

The YOLO v8n-seg model, which integrates the structures of FPN and PAN (see Figure 12), enhances the capture of top-level semantics, thereby improving target recognition. Nevertheless, there may be challenges in the detection of small-sized targets. This is due to the potential for position information from lower layers to be overlooked during the feature fusion process, resulting in a loss of effectiveness in the detection of these targets. To address this limitation, this research introduces the BiFPN structure, namely the bidirectional feature pyramid network, as a more advanced technique for feature fusion. In contrast to traditional approaches that rely on adjacent feature fusion, the BiFPN employs a bidirectional cross-scale feature fusion strategy and further optimizes the integration efficiency of features through a weighted approach. This design not only enhances the flow of information between features at different levels but also improves the model’s accuracy in recognizing targets of various sizes. Especially for small-sized targets, BiFPN effectively retains crucial positional information while incorporating high-level semantic information, achieving a higher level of feature fusion. Through this efficient and flexible method of feature fusion, the model can acquire more comprehensive contextual information, thereby significantly enhancing the overall precision of target detection [55].

Three key modifications were made to the YOLOv8n-seg model based on the modules. First, RepVit was introduced in the ‘head’ part of the model, immediately after processing the P5 feature layer and before the final feature fusion, to refine the P5 feature layer, which represents the deepest feature. Second, during the P4 processing stage of the model’s backbone, the ghost convolutional layer was used to replace the standard CBS layer found in the original model. Last, instead of using simple concatenation to merge feature maps across different scales, a BiFPN layer was introduced. This modification enhances the logical flow of information and establishes more robust causal connections between different feature representations.

#### 4.3.2. Separation of Main Stem and Lateral Stems

**Figure 13:**
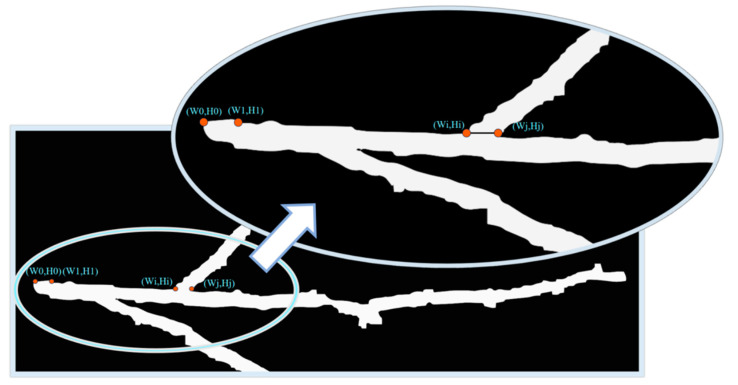
Schematic diagram of the main stem and branch splitting algorithm.

The main stem and branch splitting algorithm called midpoint coordinate algorithm is shown in Figure 13. To begin, examine the image from left to right, column by column, and identify the first non-zero-pixel point, which is a point with a pixel value other than zero. This point will be referred to as A0 and will have coordinates (W0, H0). Next, increase the horizontal coordinate W0 of A0 by 60 pixels to obtain a new horizontal coordinate W1 = W0 + 60. And W0 plus 60 is because at the root of the main stem, due to the uneven cut during harvesting, it may lead to some errors in identification, and the addition of 60 avoids the cut portion. Then, using W1 as the horizontal coordinate, search from top to bottom until you encounter the first non-zero-pixel point, which will be labeled A1 and will have coordinates (W1, H1). Point A1 is redefined as the new starting point. Then, increase the horizontal coordinate W1 of A1 by 2-pixel units in the horizontal direction, denoted as W2 = W1 + 2. Search for non-zero-pixel points in the vertical direction within the range of H1–3 to H1. If a zero-pixel point is encountered within this range, continue moving the horizontal coordinate to the right by 2 units until a non-zero-pixel point is found. Once a non-zero-pixel point is found within this range, the coordinates of the current position (Wi, Hi) are recorded. The search is then carried out to the right on the same longitudinal coordinate Hi until the first zero-pixel point (Wj, Hj) is encountered, where Hi = Hj. Finally, the white pixel point between these two points is modified to black, i.e., the pixel value is set to 0. This processing efficiently separates the main stem from the lateral stems in the image.

#### 4.3.3. Extraction of Main Stem and Branch Contours

In the initial stages of preprocessing RGB images, the conversion to the HSV (Hue, Saturation, Value) color space was undertaken to enhance the differentiation between soybean plants and their background. Analysis of the three HSV channels demonstrated that the S-channel (Saturation) provided the most pronounced contrast, effectively distinguishing the soybean plants from their surroundings. Leveraging this S-channel image, soybean plants were successfully extracted, though some residual background noise persisted.

To address this issue, a region area threshold was applied, which effectively filtered out smaller, irrelevant regions of noise and produced a clean, well-defined binarized image of the plant. This step was crucial in isolating the soybean plants from extraneous background elements.

Following binarization, semantic segmentation was employed specifically for the soybean pods. This process involved generating a detailed mask of the pods, which, when removed, revealed the underlying structure of the plant, including the main stem and branching system. By focusing on these refined images, the analysis was able to accurately expose and assess the plant’s main stem and branches, facilitating more precise and meaningful evaluation of the plant’s structural characteristics.

#### 4.3.4. Stem Length Acquisition Algorithm

A* (A-Star) search [56] is one of the most efficient directed search methods for finding the shortest path in a static network. The A* algorithm’s core lies in the design of its valuation function, defined as f(n)=g(n)+h(n). The function’s g(n) component represents the cost from the starting search point to the current point, while h(n) represents the valuation of the current node to the target node. The design of h(n) directly affects the algorithm’s performance, and a well-designed h(n) can make the algorithm more efficient in finding the optimal solution. In this study, the stem length of soybean plants was calculated by determining the shortest distance between two points in an image pixel. Branch length was measured as the distance from the start of the branch to its intersection with the main stem. A binarized image processing method was employed to create the pixel mapping, with obstacle areas represented in black and passable paths represented in white. By identifying the shortest passable path between two specific points, the length of the stem can be effectively determined. Furthermore, semantic segmentation can also be employed to create pixel mapping [7].

**Figure 14:**
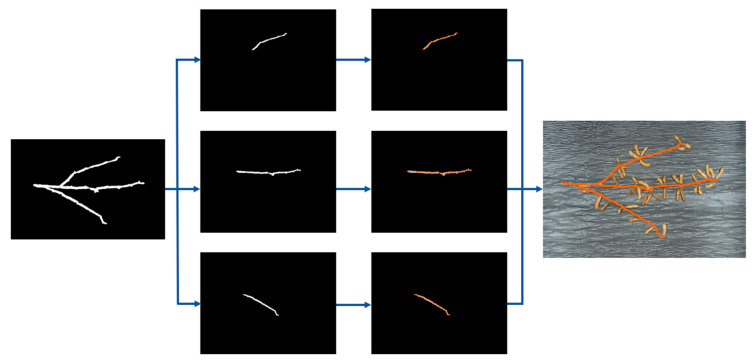
Flowchart for obtaining stem length and branch length: the orange area refers to the center of each column of white pixels.

While the A* algorithm can be employed to measure the stem length of soybean plants, it faces significant challenges due to its computational intensity and time-consuming nature. Additionally, the algorithm necessitates a precise identification of the endpoints of each stem, which becomes increasingly laborious with complex soybean images. To address these issues, the MCA was developed for measuring stem length. As depicted in Figure 14, the approach involves processing the main and lateral stems separately. Length measurements are determined by identifying the midpoint coordinates of the white pixels within each column of the image and averaging these values. The overall length of the soybean plant is then calculated by computing the Euclidean distance between these midpoints. This method significantly improves efficiency and precision by obviating the need to pinpoint the exact starting and ending coordinates of the stems, thus reducing computational demands and accelerating the processing time.

The y-coordinate of middle point in column i, $y_{i}$, can be calculated using the following equation, where $Y_{ij}$ is the vertical coordinate of the jth white pixel in the ith column.

(2) $$y_{i}=\frac{1}{N_{i}}\;\sum_{j=Y_{i1}}^{j=Y_{in}}Y_{ij}\;,$$

where $N_{i}$ is the total number of white pixels in column i. $Y_{i1}$ is the vertical coordinate of the first white pixel point in the first column, and $Y_{in}$ is the vertical coordinate of the last white pixel point in the ith column.

To calculate the Euclidean distance between these midpoint coordinates, the following formula can be used:

The Euclidean distance between two midpoint coordinates $M_{i}=(x_{i},y_{i})$ and $M_{i+1}=(x_{i+1},y_{i+1})$, which are the midpoint coordinates of neighboring columns, can be calculated using the following formula:

(3) $$D_{i,i+1}=\sqrt{{\left(x_{i+1}-x_{i}\right)}^{2}+{\left(y_{i+1}-y_{i}\right)}^{2}\;},$$

(4) $$D_{total}=\sum_{i=x_{1}}^{i=x_{m}}D_{i,i+1}$$

where $x_{1}$ is the horizontal coordinate of the white pixel point appearing in the first column. $x_{m}$ is the horizontal coordinate of the white pixel point appearing in the last column.

The stem’s total length is calculated by finding the Euclidean distance between each pair of neighboring midpoint coordinates, where $x_{i},y_{i}$, and $x_{i+1},y_{i+1}$ represent the horizontal and vertical coordinates of the two adjacent midpoint coordinates.

### 4.4. Equipment

The entire process of model training and validation was implemented on a personal computer (CPU: Intel^®^ Core™ i9 14900K @6.00 GHz; GPU: NVIDIA GeForce RTX 4080 16 G). The software environment includes the CUDA 12.1 acceleration package, Python 3.9, and Pytorch 2.1.2 for generalized deep learning functionality [57]. Key training parameters are detailed in Table 5, which includes an input image size of 640 × 640 pixels, a batch size of 16, and a training duration of 100 epochs. The maximum learning rate was set to 0.001, with Stochastic Gradient Descent (SGD) as the optimizer and a weight decay of 0.0005 to mitigate overfitting. The training process was executed with 32 threads to enhance computational efficiency. The environment is configured as shown in Table 6. The hyperparameter settings were continuously adjusted and this set of hyperparameters performed best in this study.

## 5. Conclusions

This study proposed a rapid and automated method for collecting phenotypic information on mature soybean plants at harvest in a laboratory setting with a simple background. The soybean plants were first segmented by instars using four YOLOv8-based models to successfully recognize pods and distinguish the number of soybeans in each pod. Subsequently, the phenotypic information of soybeans was obtained. The YOLOv8-Repvit model demonstrated superior performance, with recognition $R^{2}\;$ coefficients of 0.96 for both pods and soybeans, and root mean square errors of 2.89 and 6.90 for pod and bean recognition, respectively. Furthermore, an algorithm designated as MCA has been put forth as a means of efficiently separating the primary stems and branches of soybean plants. The algorithm links the white pixels representing stems in each column of a binary image to create curves representing plant structures, thereby reducing computational time and spatial complexity. This study presents an efficient technological approach to accurately measure the phenotypic characteristics of soybean plants, thereby establishing a technological foundation for obtaining the phenotypes of densely overlapping and zoned mature soybean plants under field conditions at harvest time. This methodology holds significant promise for advancing breeding practices and the development of smart agriculture.

## Figures and Tables

**Table 1:** The summary of results of the correlation analysis.

Prediction Objects	Models	*R* ^2^	RMSE
Number of pods per plant	YOLOv8	0.87	5.33
	YOLOv8-Repvit	0.96	2.89
	YOLOv8-Ghost	0.87	5.64
	YOLOv8-Bifpn	0.61	8.11
Number of beans per plant	YOLOv8	0.90	11.80
	YOLOv8-Repvit	0.96	6.90
	YOLOv8-Ghost	0.90	12.50
	YOLOv8-Bifpn	0.65	17.33
Stem and branch length	MCA	0.96	3.42

**Table 2:** The summary of results of the 4 deep learning models based on YOLOv8.

Model	Box (P)	Box (R)	Box (mAP50)	Mask (P)	Mask (R)	Mask (mAP50)	Prediction Time
YOLOv8	0.785	0.802	0.848	0.786	0.797	0.846	7.6 ms
YOLOv8-Repvit	0.786	0.805	0.857	0.783	0.810	0.856	7.7 ms
YOLOv8-Ghost	0.796	0.789	0.854	0.795	0.785	0.849	7.8 ms
YOLOv8-Bifpn	0.781	0.814	0.855	0.780	0.813	0.853	7.9 ms

**Table 4:** Experiment dataset setup and descriptions.

Dataset Type	Data Volume	Description
Original dataset	442 images	Contains images of 442 soybean plants to enhance model generalization.
Extended dataset	2210 images	Expanded to 2210 images through data enhancement techniques (brightness adjustment, level flipping, noise addition, and image panning)
Training dataset	1545 images	70% of the extended dataset was used to train the model.
Validation dataset	665 images	30% of the extended dataset was used to validate the model.
Test dataset	200 images	An additional 200 soybean plant images were collected to test the four YOLOv8-based models and the proposed stem and branch length extraction methods.
Total number of labeled pods	12,110	The total number of all labeled beanings. Soybeans were categorized by the number of grains as 1, 2, or 3, while four or more beans were categorized as 3 beans because the percentage was less than 0.5%. After data augmentation, a total of 60,550 soybean pods were obtained.
Total number of labeled plants	442 plants	Total number of labeled plants used for training and validation.

**Table 5:** Training parameter settings for four YOLOv8-based deep learning models.

Hyperparameter	Value
Input image size	640 × 640
Batch size	16
Epoch	100
Maximum learning rate	0.001
Optimizer	SGD
Weight decay	0.0005
Thread count	32

**Table 6:** Configuration of the training environment.

Name	Information
CPU	Intel^®^ Core™ i9 14900K @6.00 GHz
GPU	NVIDIA GeForce RTX 4080 16 G
Operating system	Windows 11
Deep learning framework	Pytorch 2.1.2 [57]
Programming language	Python 3.9
Integrated development environment	VScode
Package management tools	Anaconda

## Data Availability

The data in this article are available upon request due to privacy.
